# Effect of Selenium on Expression of Apoptosis-Related Genes in Cryomedia of Mice Ovary after Vitrification

**DOI:** 10.1155/2020/5389731

**Published:** 2020-09-23

**Authors:** Reza Nori-Garavand, Maryam Hormozi, Leila Narimani, Nasim Beigi Boroujeni, Asghar Rajabzadeh, Leila Zarei, Masoud Beigi Boroujeni, Mandana Beigi Boroujeni

**Affiliations:** ^1^Department of Anatomical Sciences, Lorestan University of Medical Sciences, Khorramabad, Iran; ^2^Razi Herbal Medicines Research Center, Lorestan University of Medical Sciences, Khorramabad, Iran; ^3^Department of Biochemistry, Lorestan University of Medical Sciences, Khorramabad, Iran; ^4^Department of Biochemistry, Branch of Tehran-Shargh, Payame Noor University, Tehran, Iran

## Abstract

**Introduction:**

Freezing of ovarian tissue is used for preservation of fertility. The freezing-thawing process is accompanied by oxidative stress and induction of apoptosis. Apoptosis is a complex process that has been studied in animal models. The present study was aimed at investigating the effect of selenium on suppression of apoptosis during vitrification-thawing process of mice ovary via studying expression of apoptosis-related genes, and also, we aimed to design statistical models for the roles of single genes and gene-gene interactions in suppression of apoptosis.

**Methods:**

A total of 10 right ovary samples from 10 mice were randomly divided into two groups of selenium treatment (at dose 5 *μ*g/ml sodium selenite, through adding to the media) and control group. Vitrification-thawing process was done according to the existed protocols. Real-time PCR was used for gene expression study. The apoptosis gene profile included *P53*, *Bax*, *Fas*, and *Bcl-2*. General linear model was applied to study single gene associations and gene-gene interactions.

**Results:**

From the studied genes, *P53* showed a significant downregulation in the selenium group in comparison to the control group (∆∆CT = 1.96; *P* = 0.013; relative expression (RE) = 0.28). *Bcl-2* showed a significant upregulation in the selenium group in comparison to the control group (∆∆CT = −2.49; *P* < 0.001; RE = 3.49). No significant result was found for other genes. According to the multiple models, *Bcl-2* showed a protective single gene association (beta = −0.33; *P* = 0.032), and *Fas*∗*Bcl-2* interaction was significantly positive (beta = 0.19; *P* = 0.036).

**Conclusion:**

Addition of selenium to cryomedia of vitrification-thawing process could reduce the apoptosis induced by freezing-thawing stress in mice ovary via downregulation of *P53* and upregulation of *Bcl-2* at transcription level. Multivariable statistical models should be performed in future researches to study biological systems.

## 1. Introduction

### 1.1. Background

Some women need to preserve their fertility for their future life. The most important reason for the necessity of fertility preservation is cancer [1]. During chemotherapy and radiotherapy, ovaries may be affected by chemical and physical cytotoxic agents [2]. To solve this problem, freezing of ovarian samples was suggested [3]. Other than ovarian samples, sperms and spermatogonial stem cells are also frozen. There are usually two freezing methods consist of slow freezing (conventional cryopreservation) and rapid freezing (vitrification or rapid cryopreservation). Freezing-thawing process is associated with oxidative stress and cell damage. Therefore, using additive antioxidants in cellular or tissue preservation media has been suggested [4].

Selenium is a biological trace element plays a key role in human metabolism. Selenium is used for synthesis of selenoproteins in the liver. This element performs its function as a component of antioxidant enzymes [5]. Other than nutritional values, selenium has an important role in human immune system [6]. Because of the antioxidant effects of selenium, it is widely used in the studies of the animal models of oxidative stress [7].

Apoptosis is a kind of programmed cell death which is usually a physiological phenomenon. However, if the microenvironment of the cells is not suitable, a pathologic apoptosis may occur [8]. Freezing-thawing damage is an example of this unsuitable environmental condition [9]. So far, many animal studies have been designed to model induction of apoptosis. For instance, chemotherapeutic agents are used for induction apoptosis in animal models [10]. In a rabbit osteoarthritis model, transaction of anterior cruciate ligament was applied for induction of apoptosis in chondrocytes [11]. Other than *in vivo* studies, animal cells and tissues are used *in vitro* for apoptosis modeling. Freezing-thawing process is a common method for induction of apoptosis in a practical condition [12, 13]. Nevertheless, the exact mechanism of apoptosis induction and apoptosis suppression by antioxidants is not completely understood.

### 1.2. Objectives

Since there was a gap in evidence on the role of supplementation of nutritional elements to cryomedia of ovarian freezing-thawing process, this study was aimed at evaluating the role of selenium supplementation on reduction of apoptosis after vitrification of mice ovarian tissue through study of apoptosis-related genes including *P53*, *Bax*, *Bcl-2*, and *Fas*. In addition, a statistical modeling was designed to study the roles of single genes and gene-gene interactions in suppression of apoptosis. Our aims were both basic and practical.

## 2. Material and Methods

### 2.1. Study Design

An experimental study was conducted with *in vitro* design. This study consisted of cellular media processing and gene expression assay.

### 2.2. Animals and Study Groups

A total of 10 balb/c mice aged 6-8 weeks were bought from an animal laboratory of Lorestan University of Medical Sciences, Khorramabad, Lorestan Province, West of Iran. After approving the study protocol by the ethics committee of Lorestan University of Medical Sciences (registration number: IR.LUMS.REC.1397.195), the animals were imported to the study according to ethical guidelines for working with laboratory animal. All the animals were kept at standard condition of light, temperature, moisture, and nutrition. Pseudopregnancy was induced using a vaginal swab. The animals were sacrificed with cervical dislocation. The corpses were kept in supine position and fixed, and the abdominal hair was shaved. In a sterile condition, midline excision was conducted from xiphoid to pubis, and then, peritoneum was excised, and abdominal viscera were removed. The right ovaries were removed by surgical pans and knife for the study. The extra tissues around the ovaries were removed under a stereomicroscope. The ovarian particles were kept in Dulbecco's modified eagle medium (DMEM) culture environment with 10% fetal bovine serum (FBS). The samples were randomly divided into two groups: selenium group and control group.

### 2.3. Sample Processing

Ethylene glycol ficoll sucrose (EGFS) media was used for vitrification process according to a protocol mentioned by Chang et al. [14]. EGFS solution was prepared in a DMEM solvent containing of 40% ethylene glycol, 30% ficoll 70, and 0.5 mole sucrose. For the selenium group, 5 *μ*g/ml sodium selenite was added to EGFS solution [15]. Then, the EGFS was filtered with a 0.45 *μ*m millipore filter and kept at -20°C. After preparation of the solutions EGFS and EGFS+Se, the ovarian samples were added to the solutions, and after dehydration, they were put in the cryovials. The dehydration process took about 5 minutes. The cryovials were put on nitrogen vapor for 30 seconds, and then, they were put at temperature -196°C in liquid nitrogen. For thawing process (rapid method), serial concentrations of sucrose solution were used as 1, 0.5, and 0.25 mole per one litter of DMEM, and the forth step was pure DMEM. The hydration process took about 5 minutes. After hydration process, the ovarian samples were kept in DMEM with 10% FBS, under CO_2_ incubator at temperature 37°C for 24 hours. During this period, the samples were washed for some times. Finally, the ovarian samples were subjected for RNA extraction and gene expression study.

### 2.4. Molecular Assay

Total RNA was extracted using a column-based RNA extraction kit (Yektatajhiz Azma, Iran). About 20-30 mg of each ovarian tissue was used. The extracted RNA was preserved in an RNAse free elution solution. A NanoDrop spectrophotometer was used to read the concentrations. Then, the extracted RNA was kept at -70°C. cDNA was synthesized with oligo dT primers using cDNA synthesis kit (Sina Clone, Iran). Primers were designed with help of NCBI database (Table 1). GAPDH was used as internal control. Gene expression was studied via real-time polymerase chain reaction (real-time PCR). SYBR green master mix was used for amplification of the genes. The amplification process was observed and documented in Rotor Gene program. Each gene of each sample was run for 3 times, and then, the best results were chosen for analysis according to the melting curves and reaction efficiencies.

### 2.5. Data Analysis

Amounts of CTs were calculated based on takeoff and amplification cycles at the exponential phase. Reaction efficiencies were obtained from REST2009 program. ∆CT amounts were calculated via CT of target gene minus CT of reference gene. ∆∆CT amounts were also calculated in which one ∆CT was for the treated group and the other ∆CT was for the control group. Relative expression (RE) was calculated with Pfaffl's efficiency calibrated method in which each ∆CT was defined as CT of sample minus CT of control for each gene (equation (1)) [16]. RE was also calculated for comparison of apoptosis stimulating genes with *Bcl-2* apoptosis inhibitory gene. For statistical analysis, independent *t*-test was used for comparison of -∆CTs between the groups, and one sample *t*-test was used for comparison of -∆∆CTs with zero (as the null hypothesis). In order to adjust the associations of the target genes and their interactions with *Bcl-2* gene, a general linear model was used, and beta coefficient with 95% confidence interval (CI) was reported. All the analyses were done before computing RE. Heat map was used to cluster the individual sample data. All the mathematical and statistical process was performed in the R 3.6.3 software (R foundation for statistical computing, Austria) [17].

(1) $$\begin{array}{c} \text{RE}=\frac{{\left(E_{\text{target}}\right)}^{-∆\text{C}{\text{T}}_{\left(\text{sample}-\text{control}\right)}}}{{\left(E_{\text{reference}}\right)}^{-∆\text{C}{\text{T}}_{\left(\text{sample}-\text{control}\right)}}}, \end{array}$$

where *E* = 2^Efficiency (%)^.

## 3. Results

After extraction of RNA, the concentration of RNA was obtained at range 69.60-79.40 ng/*μ*l. Using 1 *μ*l of cDNA and 1 *μ*l of 10 pmol primers resulted in successful amplification with one melting peak at a suitable temperature. From the repetitive reactions, those with better reaction efficiencies were selected. REs were calculated based on ∆CT_(sample-control)_ amounts adjusted with the efficiencies while statistical analyses (and calculation of P values) were done based on ∆CT_(target-reference)_ and ∆∆CT amounts (Tables 2 and 3). Our sample size had about 79% power to detect at least two amplification cycle differences with standard deviation of one cycle at the point of 5% type one error.

**Figure 1:**
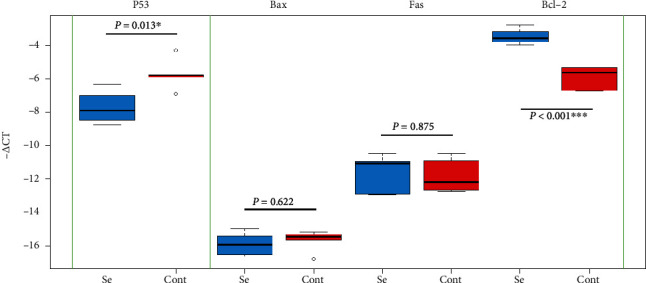
Gene expression comparison in selenium (Se) and control (Cont) groups (independent *t*-test).

**Figure 2:**
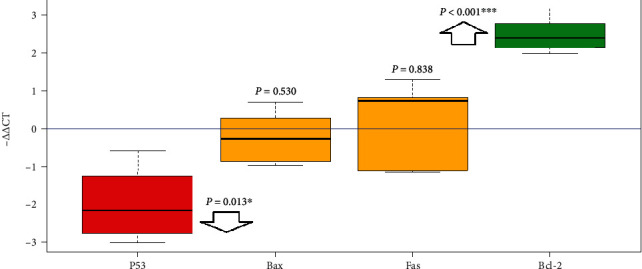
Gene expression of the target genes if the treated group calibrated with the control group (one sample *t*-test).

**Figure 3:**
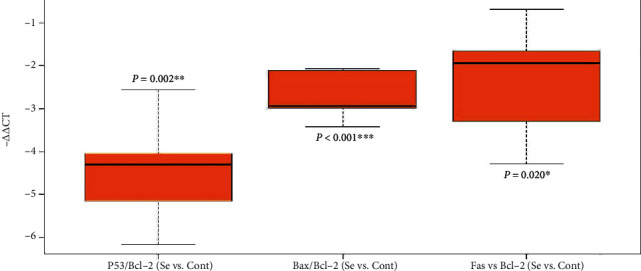
Gene expression of the target genes per expression Bcl-2 in the treated group calibrated with the control group (one sample *t*-test).

**Figure 4:**
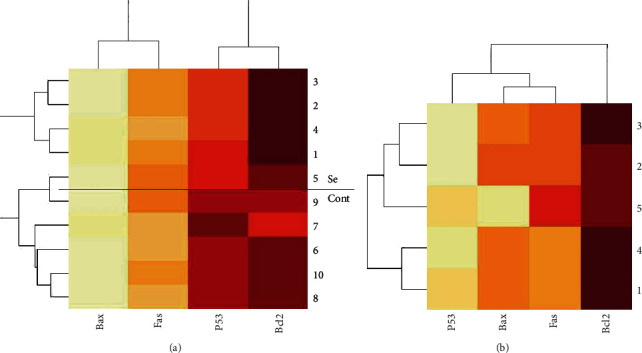
Clustering gene expression in individual samples. (a) -∆CTs in selenium (Se) and control (Cont) groups. (b) -∆∆CTs in each treated sample.

Among the four target genes, *P53* showed a significant downregulation (*P* = 0.013; RE = 0.28), and *Bcl-2* showed a significant upregulation (*P* < 0.001; RE = 3.49) in the treated group. The results of *Bax* and *Fas* were nonsignificant and inconclusive (Table 2) (Figures 1 and 2). In order to assay overall status of apoptosis with a gene expression approach, expression of target genes per *Bcl-2* was studied. All the target genes showed downregulation in comparison to *Bcl-2* antiapoptotic gene that the most downregulation was for *P53* (RE = 0.08) followed by *Bax* (RE = 0.25) and *Fas* (RE = 0.30) (Table 3) (Figure 3). Clustering of the individual samples showed similar results in the samples, except for sample 5 of the selenium group which was clustered in the cluster of the control group. Clustering of the genes showed that in condition of apoptosis, *Bcl-2* had more expression followed by *P53*, *Fas*, and *Bax*, of course without considering better efficiency of *P53* in our study (Figure 4).

**Figure 5:**
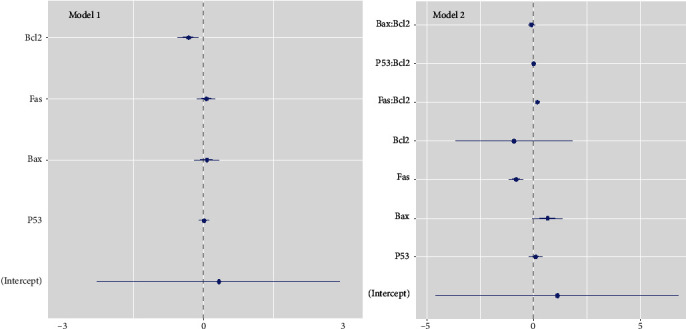
Coefficient plot of general linear model for +∆CTs of the genes. Model 1: multiple adjusted model. Model 2: multiple adjusted model with interaction of target genes with Bcl-2.

The general linear model was used to reach a per se association of each gene without the confounding effect of other genes. Accordingly, each unit of increase in ∆CT (i.e., downregulation up to two folds) for *Bcl-2* was independently associated with about 33% reduction in the probability of being in the selenium-treated group (beta = −0.33; *P* = 0.032). No significant association was found for other genes (Table 4, model 1) (Figure 5, model 1). Since *Bcl-2* had a better per se association and also was the only antiapoptotic gene, its interactions with the other target genes were also imported to the model. Accordingly, *Fas*∗*Bcl-2* interaction was positive (beta = 0.19; *P* = 0.036). No significant result was obtained for interactions *Bax*∗*Bcl-2* and *P53*∗*Bcl-2*. Interestingly, among the single genes, the association of *Fas* became significant (beta = −0.81; *P* = 0.045), and *Bcl-2* lost its significance (Table 4, model 2) (Figure 5, model 2). Further addition of other possible interactions to the model resulted in loss of significance or software error due to low number of observations (data not shown).

## 4. Discussion

### 4.1. Interpretation

The aim of this study was to investigate mRNA expression profile of apoptosis-related genes in an animal tissue model of apoptosis induction by vitrification. This study consisted of the profile response to selenium administration as an antioxidant and investigation of gene-gene interactions in the mentioned profile. The practical aim was to reach a model for fertility preservation in conditions of different human pathologies such as cancers in young women.

Among the investigated genes, changes in the expression of *P53* and *Bcl-2* were statistically significant. Other than statistical analysis based on the number of PCR cycles (i.e., ∆CT and ∆∆CT), efficiency calibrated method approved the associations of *P53* and *Bcl-2* (95% CIs of their REs had a remarkable distance from RE = 1 as a null hypothesis). In addition, proportions of *P53/Bcl-2*, *Bax/Bcl-2*, and *Fas/Bcl-2* mRNA expression were downregulated in the treated group as a manifestation of the general status of apoptosis at transcription level. It demonstrated that in biological systems, proportions of the levels of biomarkers might be more important than the pure levels to perform their biological functions.

Multivariable and multivariate analyses are a way for a better discovery and interpretation of biological systems. According to the heat map, the individual samples were clustered in their real groups except one sample of the selenium group that was clustered as the final branch of the control group. The clustering of the genes demonstrated that even in condition of apoptosis *Bcl-2* had further expression and in response to antiapoptotic agents its interval with apoptotic genes is increased. According to the general linear models, only *Bcl-2* had an independent association with the groups of study (in favor of being in the control group, i.e., antiapoptotic effect). After adjusting the model with the interactions of *Bcl-2*, the only significant associations were for *Fas* (in favor of being in the control group, i.e., antiapoptotic effect) and *Fas*∗*Bcl-2* interaction (positive interaction, i.e., they help each other for upregulation). It showed that either *Fas* had a secret effect on upregulation of *Bcl-2*, or its secret upregulation was due to a compensatory effect in response to *Bcl-2* upregulation. It was worth noting that the interaction *P53*∗*Bcl-2* did not have significant interaction in spite of their significant ratio of expression.

*P53* is a tumor suppressor gene involved in both intrinsic and extrinsic pathways of apoptosis. Therefore, downregulation of *P53* is associated with reduction in apoptosis [18, 19]. Bcl-2 is an antiapoptotic protein, and therefore, upregulation of *Bcl-2* is associated with suppression of apoptosis [20]. Proportion of Bax/Bcl-2 is a widely used marker to study apoptosis. Downregulation of this proportion at transcriptome level may indicate downregulation of apoptosis. Das et al. (2007) and Ghiasi et al. (2016) used this ratio via real-time PCR to detect apoptosis [21, 22]. However, in our study, the mRNA ratio *P53/Bcl-2* was more dominant, and the significant interaction was for *Fas*∗*Bcl-2*.

In general, our study showed an antiapoptotic effect for selenium. The antiapoptotic effect of selenium may be resulted from its antioxidant effects [23]. A controversial hypothesis is that anticancer effect of selenium is through induction of apoptosis [6]. In fact, selenium participates in DNA repair, and it is resulted from helping antioxidant enzymes [24].

### 4.2. Previous Literature

Agrawal et al. (2006) reported that during freezing-thawing process reactive oxygen species (ROS) were increased and induced apoptosis [25]. It has been shown that the effect of ROS on induction of apoptosis is mainly through activation of P53 [26]. Since in the present study *P53* was downregulated, it seems that protective effect of selenium may be due to its antioxidant effects. Rimon et al. (2005) believed that apoptosis is the final response of cell to inappropriate conditions, and therefore, apoptosis study is a suitable tool in stressful conditions of freezing protocols [27].

A lot of researches have been performed on freezing media of ovary and sperm samples. Slaweta et al. (1988) had studied the relationship between selenium content of bull semen with sperm motility after freezing-thawing damage. They found that lower selenium content was associated with lower sperm motility [28]. Dorostkar et al. (2012) studied the effect of selenium additive on sperm characteristics in water buffaloes before and after freezing, and they reached a better sperm quality [29]. Rezaeian et al. (2016) studied the effects of selenium (at dose 5 *μ*g/ml) on human sperm parameters after freezing-thawing process. They found a better sperm quality and suggested selenium supplementation for use in laboratories of infertility clinics [15]. Although there were many studies on the role of selenium in male reproduction system, there was not a study on the role of selenium supplementation in freezing-thawing process of ovary samples via studying apoptosis-related genes. It was the novelty of our study in comparison to the literature. Bozkurt et al. (2012) had found a protective effect for selenium on ischemia reperfusion injury in rat ovary via studying oxidative stress indices [30]. Brito et al. (2013) showed protective effects of selenium in combination with vitamin E on ovarian samples of monkey via investigating expression of superoxide dismutase 1 gene [31]. In general, protective effect of selenium has been observed in many studies like our study.

### 4.3. Limitations

Lack of study at proteomic level was the most important limitation of our study. Also, frozen oocytes should be studied in future. Although our sample size had an approximately suitable power for univariable analyses like other animal studies, our multiple models needed further observations to study all the possible interactions with enough power. However, our models were fit (low residual standard error) for individual predictions.

## 5. Conclusion

Addition of selenium at dose 5 *μ*g/ml of sodium selenite to cryomedia of vitrification-thawing process could reduce the apoptosis induced by freezing-thawing stress in mice ovary. This effect was thrown downregulation of *P53* and upregulation of *Bcl-2* at transcription level. According to the multiple models, *Bcl-2* showed a protective single gene association, and *Fas*∗*Bcl-2* interaction was significantly positive. This study can be performed on human ovary samples in the patients who are candidate for wedge sample or whole ovary freezing regarding ethical and legal issues. Multivariable statistical models should be performed in future researches to study biological systems.

## Figures and Tables

**Table 1:** Primer sequences for amplification of cDNA.

Gene	Primer sequence (5′-3′)	Product length (bp)
GAPDH	Forward: CAATGTGTCCGTCGTGGATCT Reverse: GTCCTCAGTGTAGCCCAAGATG	208
P53	Forward: GTTTCCTCTTGGGCTTAGGG Reverse: CTTCTGTACGGCGGTCTCTC	255
Bax	Forward: CGAGCTGATCAGAACCATCA Reverse: GAAAAATGCCTTTCCCCTTC	277
Fas	Forward: GAGAATTGCTGAAGACATGACAATCC Reverse: GTAGTTTTCACTCCAGACATTGTCC	314
Bcl-2	Forward: TAAGCTGTCACAGAGGGGCT Reverse: TGAAGAGTTCCTCCACCACC	344

**Table 2:** Expressions of the target genes (selenium group vs. control group).

Gene	∆∆CT (95% CI)	One sample *P*	Two sample *P*	Efficiency	RE (95% CI)
GAPDH	Reference			0.702	
P53	1.96 (0.69, 3.22)	0.013^∗^	0.013^∗^	0.921	0.28 (0.16, 0.47)
Bax	0.22 (-0.66, 1.11)	0.530	0.622	0.728	0.88 (0.78, 1.00)
Fas	-0.11 (-1.56, 1.33)	0.838	0.875	0.707	1.06 (0.68, 1.63)
Bcl-2	-2.49 (-3.09, -1.89)	<0.001^∗∗∗^	<0.001^∗∗∗^	0.734	3.49 (3.22, 3.78)

^∗^Significant at 0.05. ^∗∗∗^Significant at 0.001. CIs of RE were computed using RE formula on lower and upper bounds of ∆CT (sample-control for each gene separately).

**Table 3:** Expression of the target genes/Bcl-2 (selenium group vs. control group).

Gene	∆∆CT (95% CI)	One sample *P*	Efficiency	RE (95% CI)
GAPDH	Reference		0.702	
Bcl-2	Reference		0.734	
P53	4.45 (2.78, 6.11)	0.002^∗∗^	0.921	0.08 (0.05, 0.12)
Bax	2.71 (1.97, 3.44)	<0.001^∗∗∗^	0.728	0.25 (0.24, 0.26)
Fas	2.37 (0.61, 4.14)	0.020^∗^	0.707	0.30 (0.21, 0.43)

^∗^Significant at 0.05. ^∗∗^Significant at 0.01. ^∗∗∗^Significant at 0.001.

**Table 4:** General linear model for multiple adjustment of the associations of the genes with being the treated group.

Model/covariate	Beta coefficient (95%, CI)	Wald test *P*	Residual SE	*R*-squared
Model 1			0.268	0.856
P53	0.01 (-0.28, 0.31)	0.913		
Bax	0.06 (-0.29, 0.42)	0.672		
Fas	0.06 (-0.20, 0.31)	0.598		
Bcl-2	-0.33 (-0.62, -0.04)	0.032^∗^		
Intercept	0.32 (-6.37, 7.01)			
Model 2			0.111	0.990
P53	0.10 (-0.60, 0.79)	0.601		
Bax	0.64 (-0.92, 2.21)	0.218		
Fas	-0.81 (-1.58, -0.05)	0.045^∗^		
Bcl-2	-0.91 (-6.80, 4.96)	0.570		
Fas∗Bcl-2	0.19 (0.03, 0.34)	0.036^∗^		
P53∗Bcl-2	-0.01 (-0.14, 0.11)	0.646		
Bax∗Bcl-2	-0.10 (-0.45, 0.25)	0.342		
Intercept	1.10 (-23.37, 25.57)			

^∗^Significant at 0.05 (it is also the interaction sign in the first column). SE: standard error.

## Data Availability

The data (raw amounts of CTs of the real-time PCR) used to support the findings of this study are available from the corresponding author upon request.
